# Mitochondrial DNA methylation and copy number predict body composition in a young female population

**DOI:** 10.1186/s12967-019-02150-9

**Published:** 2019-11-28

**Authors:** Laura Bordoni, Vanessa Smerilli, Cinzia Nasuti, Rosita Gabbianelli

**Affiliations:** Unit of Molecular Biology, School of Pharmacy, University of Camerino, Via Gentile III da Varano, 62032 Camerino, MC Italy

**Keywords:** Mitochondrial epigenetics, DNA methylation, Buccal swabs, Body composition

## Abstract

**Background:**

Since both genomic and environmental factors are involved in obesity etiology, several studies about the influence of adiposity on both nuclear DNA and mitochondrial DNA methylation patterns have been carried out. Nevertheless, few evidences exploring the usage of buccal swab samples to study mitochondrial DNA epigenetics can be found in literature.

**Methods:**

In this study, mitochondrial DNA from buccal swabs collected from a young Caucasian population (n = 69) have been used to examine potential correlation between mitochondrial DNA copy number and methylation with body composition (BMI, WHtR and bioimpedance measurements).

**Results:**

A negative correlation between mitochondrial DNA copy number and BMI was measured in females (p = 0.028), but not in males. The mean percentage of D-loop methylation is significantly higher in overweight than in lean female subjects (p = 0.003), and a specific CpG located in the D-loop shows per se an association with impaired body composition (p = 0.004). Body composition impairment is predicted by a combined variable including mtDNA copy number and the D-loop methylation (AUC = 0.785; p = 0.009).

**Conclusions:**

This study corroborates the hypothesis that mitochondrial DNA carries relevant information about body composition. However, wider investigations able to validate the usage of mtDNA methylation from buccal swabs as a biomarker are warranted.

## Background

Obesity is a well-known risk factor for numerous chronic pathologies, including cardiovascular diseases [1] and cancer [2]. Overweight and obesity in children and adolescents represent a major burden in the modern global society, as their prevalence in recent decades has increased worldwide [3–6], and it has been demonstrated that early life obesity is a precursor to obesity in adulthood [7].

Since adiposity and obesity result from the interaction of both genetic background and environmental factors from very early in life, epigenetic processes have been indexed as potential mediators that may fill the gap in the understanding of etiology of this multifactorial pathology [8, 9]. Despite promising results and increasing efforts in this field, the epigenetic basis of obesity remains largely unknown, in particular in early developmental stages, such as childhood and adolescence [10].

DNA methylation is one of the most-studied heritable epigenetic marker able to regulate gene expression without changing the primary DNA sequence, and which can be modified by lifestyle and environmental factors [11, 12]. Several studies have described DNA methylation patterns associated with obesity [13, 14]. Most of them investigated the epigenome of genomic DNA from peripheral blood cells [15] or tissues with specific metabolic roles (i.e. subcutaneous fat [16]); some others changes can be investigated in genomic DNA from buccal swabs [17], as it is a non-invasive, easily accessible sample, whose usage in epidemiological (and epigenetic) studies is spreading [18].

Recently, it has been demonstrated that methylation marks can be detected also in the mitochondrial DNA (mtDNA) [19], but the debate about their existence and biological meaning is still ongoing. Because of its role at the interface between environmental exposure, genomics and biochemistry of energy balance, this novel epigenetic mark has been addressed to be a next generation biomarker of exposure and disease [20]. Not only mitochondrial epigenetics [21] but also mtDNA copy number can play a relevant role in numerous health and disease conditions [22–27]. Indeed, each mitochondrion contains 2–10 mitochondrial DNA copies [24] and it has been demonstrated, for instance, that high mtDNA copy number in peripheral blood is associated with higher cognition in elderly women [28], while decreased mtDNA content precedes the onset of non-insulin-dependent diabetes mellitus [29]. Interestingly, numerous studies identified that differences of mtDNA copy number are gender specific in both animal models and humans [30–32].

Despite growing interest on this topic, few data are available about the usage of buccal swabs to study mtDNA methylation and copy number in obesity [33]. Environmental epidemiology and public health researchers often rely on peripheral biological sources as they do not have access to specific target tissues related to complex diseases. Thus, in a context concerning vulnerable individuals such as children, buccal swabs and saliva samples could be useful samples with reduced discomfort, able to increase participation and improve overall feasibility of the investigation [17, 18, 34–36]. In this respect, the aim of this study is to investigate if body composition affects copy number and methylation of mtDNA extracted from buccal swab samples in a cohort of young Caucasian individuals.

## Materials and methods

### Study design and sampling

This study included 69 children and adolescents (35 males and 34 females) recruited during a sport medicine check-up. As described in a previous study [37], all the recruited subjects regularly play sports, from 2 to maximum 4 times a week. The level of physical activity of the studied population is estimated to be moderate (3–6 metabolic equivalents of task, METs), according to CDC (Centers for Disease Control and Prevention, USA) guidelines. The sports practiced are heterogeneous but characterized by alternation of aerobic and anaerobic phases (i.e. basket, volleyball, soccer, water polo, judo, figure skating, tennis, artistic gymnastic). Moreover, it must be considered that the participants are active subjects, but not agonist players. The recruitment occurred on a voluntary basis and parental consent was obtained from all participants. Exclusion criteria were (a) current or previous occurrence of metabolic or digestive disease (except for appendectomy) or kidney disease; (b) being pregnant or breastfeeding. The study protocol has been approved by the general direction of Area Vasta 2, Jesi, Marche (Italy), in accordance with the Declaration of Helsinki in its revised edition and with international and local regulatory requirements. The subset of analyzed buccal swab samples was limited by the amount of DNA requested for the mtDNA isolation procedures explained as follow. As sex-dependent effects on mtDNA have been previously observed [30–32], the investigations were carried out in male and female population separately.

### Body composition assessment

Data concerning age, height, weight (Wunder A150) and waist circumference (WC) (Hoechst mass roll fix tape) of recruited subjects were collected by trained evaluators according to standard protocols, as explained in the previous paper [37]. Body mass index (BMI) was calculated as weight divided by height squared, while waist to height ratio (WHtR) was determined by dividing the waist circumference for their height, both measured in the same units. Subjects with the BMI percentile > 85th according to sex and age were classified as overweight (as percentiles represents the gold standard to define overweight in children), in agreement with the IOTF cut-offs [38]. Bioelectrical impedance analysis (BIA) was carried out using BIA (BIA Akern 101, Akern s.r.l), as previously described [39]. Data about FM percentage (FM%), body cellular mass (BCM), and phase angle (PhA) were collected. BCM was normalized for height by calculating the BCM index (BCMI) [18]. The PhA [40] was derived as the arctangent of the reactance-to-resistance ratio that was directly measured through BIA analysis. PhA is one of the most relevant impedance parameter as it correlates with various indexes of functional and nutritional status [41–43].

### DNA isolation and mtDNA copy number assessment

Genomic DNA was extracted from buccal swabs through isopropanol precipitation. MtDNA copy number was assessed by qPCR method (as previously described [44, 45]) using primers specific for mtDNA (mtpair21 fw-AATCCAAGCCTACGTTTTCACA; mtpair21 rv-AGTATGAGGAGCGTTATGGAGT) and normalizing for the amount of genomic DNA used in each reaction by using primers specific for gDNA (18s fw-GCAATTATTCCCCATGAACG; 18s-rv GGGACTTAATCAACGCAAGC). qRT reaction was performed using Takara TB Green™ Premix Ex Taq™ II (Takara) by using CFX-96 (Biorad).

### Purification of mtDNA and evaluation of D-loop methylation

MtDNA was isolated from nuclear DNA (ncDNA) according to the technique previously reported by Jayaprakash and colleagues [44] referred as Mseek, which is an innovative and efficient method based on the selective digestion of ncDNA without degrading the circular mtDNA. Briefly, 4 μg of gDNA were digested with the Exonuclease V (M0345S, NEB) at 37 °C for 48 h. After the heat inactivation of the enzyme (70° for 30 min), the DNA was purified by using Agencourt AMPure XP magnetic beads (Beckman coulter) according to the manufacturer’s protocol. A second digestion with Exonuclease V was then performed in the same conditions and, after the purification, the isolated mtDNA was tested for ncDNA contamination with the same primers used to quantify the mtDNA, as described in the previous paragraph. MtDNA was considered pure when amplification products for ncDNA were obtained at cycles higher than 30 and a delta of at minimum of 8 cycles between nuclear and mitochondrial Ct could be measured.

MtDNA methylation of two areas of the D-loop was assessed by bisulphite pyrosequencing. MtDNA was converted by bisulphite using the EZ DNA methylation gold kit (Zymo research), according to the manufacturer’s protocol. The following primers were used to amplify the selected regions: MT2 fw–GTGTATTGTTTTGAGGAGGTAAGT; MT2 rv-biotin-CACTCCCATACTACTAATCTCATCAAT; MT2 seq-TTTTTGGGGTTTGGT; MT20 fw-TGGATGATTTTTTTTAGATAGGGGTTTT; MT20 rv-biotin- CAATTCACTTTAACTACCCCCAAATA; MT20 seq-AGGGGTTTTTTGATTATTATT. These two regions were selected since they previously showed highest methylation levels in mtDNA [47].

### Statistical analysis

Data were analyzed using SPSS (SPSS Inc., Chicago, IL, USA). Kolmogorov–Smirnov test was used to assess normality of distributions. Pearson correlation was tested to demonstrate significant correlations among normally distributed variables. A logarithmic transformation was used to normalize variables whenever it was possible. Spearman correlation was used with the same aim for not normally distributed parameters. General linear models were used to detect associations among categorical and continuous variables. ROC curves were calculated to test the ability of different variables to predict overweight. The combined predicted probability was calculated according to a binary logistic regression model. Two-tailed p values are reported, and differences were considered significant when p values were < 0.05.

## Results

### Descriptive statistics

Because of technical problems in mtDNA isolation, 57 samples out of 69 were analyzed.

Kolmogorov–Smirnov test revealed that all the primary variables were normally distributed except for the variable describing mtDNA copy number (that was normalized by logarithmic transformation) and the combined predicted probability calculated to test the combined effect of mtDNA copy number and mtDNA methylation (CP*MH). Descriptive statistics for the total population stratified by sex are shown in Table 1.

**Table 1:** Descriptive statistics for the total population divided by gender

	Males (n = 27)				Females (n = 30)			
	Min	Max	Mean	SD	Min	Max	Mean	SD
Age	8.00	17.00	12.70	2.52	7.00	17.00	11.20	2.94
Weight	21.70	117.80	51.38	19.49	21.40	82.00	43.87	16.48
Height	120.00	184.00	158.00	17.31	119.00	173.50	146.36	16.46
BMI	13.86	34.79	19.86	4.09	14.54	28.37	19.74	4.01
WC	51.00	98.00	68.55	9.03	51.00	85.00	63.72	9.47
WHtR	0.37	0.53	0.43	0.03	0.37	0.53	0.43	0.04
FM (%)	10.44	35.61	21.95	6.51	11.14	41.84	27.10	6.49
BCM	7.78	41.64	20.66	8.17	6.94	29.20	15.61	6.18
BCMI	5.00	12.30	7.91	1.74	4.60	9.70	6.98	1.51
PhA	4.50	6.70	5.51	0.63	3.80	6.60	5.14	0.67
BMI/PhA	2.70	6.10	3.61	0.71	2.90	5.37	3.86	0.75

*BMI* body mass index, *WC* waist circumference, *WHtR* waist to height ration, *FM* fat mass, *BCM* body cellular mass, *BCMI* BCM index, *PhA* phase angle

### MtDNA copy number is correlated with body composition in females, but not in males

Data analysis of relative amount of mtDNA copy number revealed higher mean values in the female than in the male population (F = 6.676; M = 3.175, p = 0.027). Age does not affect mtDNA copy number neither in the male (Pearson correlation = 0.017, p > 0.05) nor in the female population (Pearson correlation = − 0.205; p > 0.05). This is expected as the analyzed population is homogeneous for age.

**Fig. 1:**
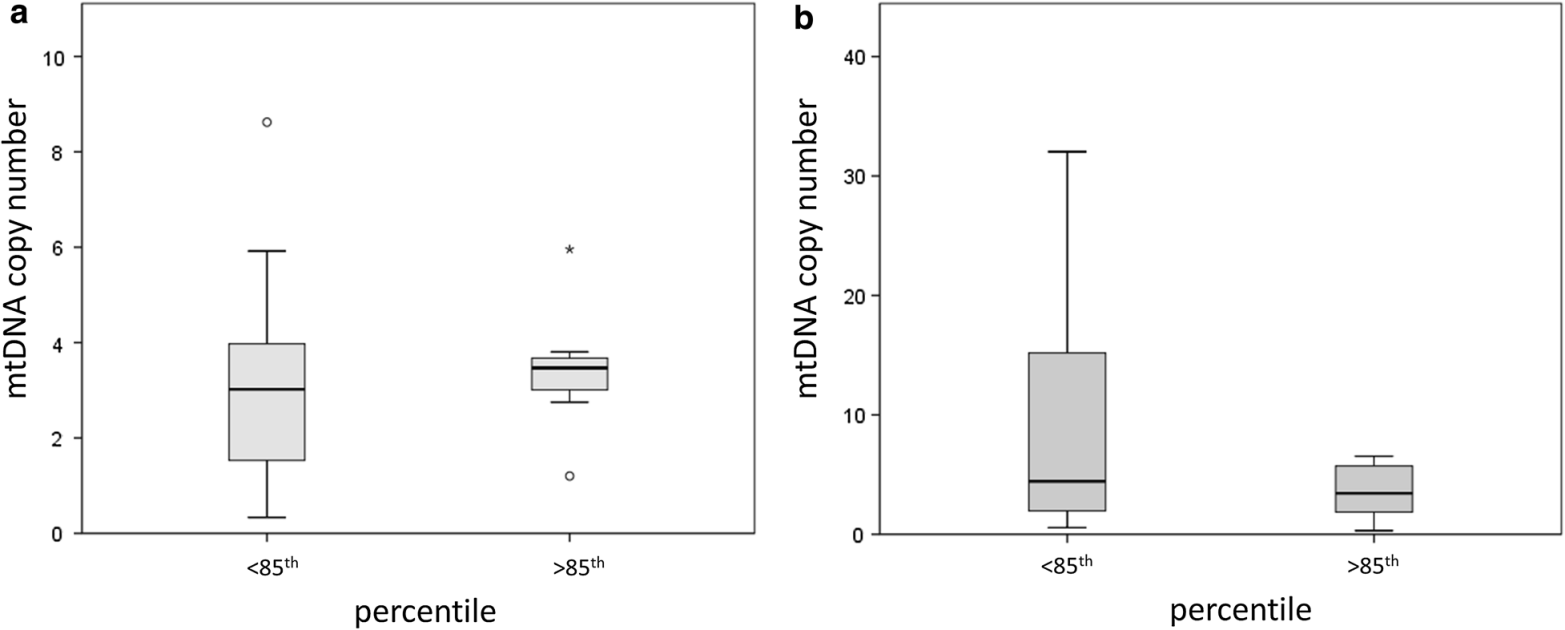
MtDNA copy number and body percentile. Box plots represents differences in mtDNA methylation in the male (**a**) and female (**b**) population divided for higher/lower than 85th percentile

Whether we divide the population for the percentiles (higher/lower than 85th), mtDNA copy number does not differ between normal weight or overweight individuals, neither in males (GLM; p > 0.05) (Fig. 1a) nor in females (GLM; p > 0.05) (Fig. 1b).

**Fig. 2:**
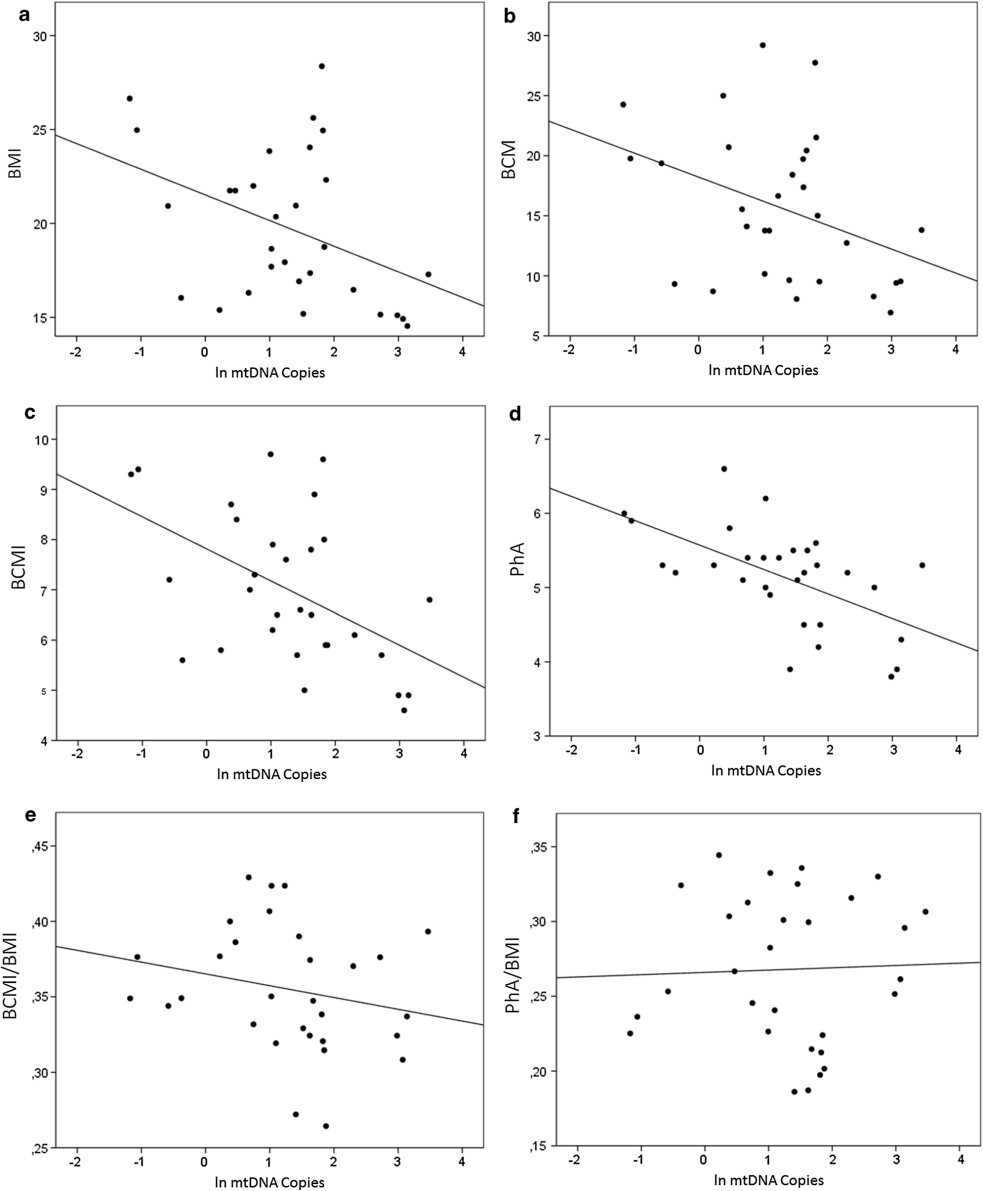
Correlation between mtDNA copy number and BMI (**a**), BCM (**b**), BCMI (**c**), PhA (**d**), BCMI/BMI (**e**), PhA/BMI (**f**) in the female population

None of the continuous variables describing body composition resulted to be correlated with mtDNA copy number in males (Pearson correlation, p > 0.05). On the other hand, a negative correlation between mtDNA copy number and BMI (Pearson correlation = − 0.402; p = 0.028) was measured in the female population (Fig. 2a).

Analyzing BIVA data about body composition, mtDNA copy number resulted to be negatively associated also to BCM (Pearson correlation = − 0.387; p = 0.035) (Fig. 2b), BCMI (Pearson correlation = -0.498; p = 0.005) (Fig. 2c) and PhA (Pearson correlation = − 0.576; p = 0.001) (Fig. 2d) indexes. However, this association is not significant if we normalize BIVA parameters for BMI; indeed, no significant association could be detected between mtDNA copy number and BCMI/BMI (Pearson correlation = − 0.221; p > 0.05) (Fig. 2e) or PhA/BMI (Pearson correlation = 0.036; p > 0.05) (Fig. 2f). This is in accordance with previous literature suggesting that BMI could act as a cofounder for BIVA parameters such as PhA measured in children and adolescents [48].

### Higher methylation of a CpG in the D-loop is associated with impaired body composition in females

Since preliminary data on mtDNA copy number suggest a correlation with body composition in females but not in males, pyrosequencing of D-loop was performed only in the subgroup of 30 female individuals. 7 CpGs located in two areas of the D-loop (MT2 and MT20) were analyzed singularly and then a mean % value of methylation has been calculated for each of the two areas. Data analysis reveals that age does not affect methylation levels in none of the analyzed CpGs, neither singularly nor as mediated value (Pearson correlation, p > 0.05).

**Fig. 3:**
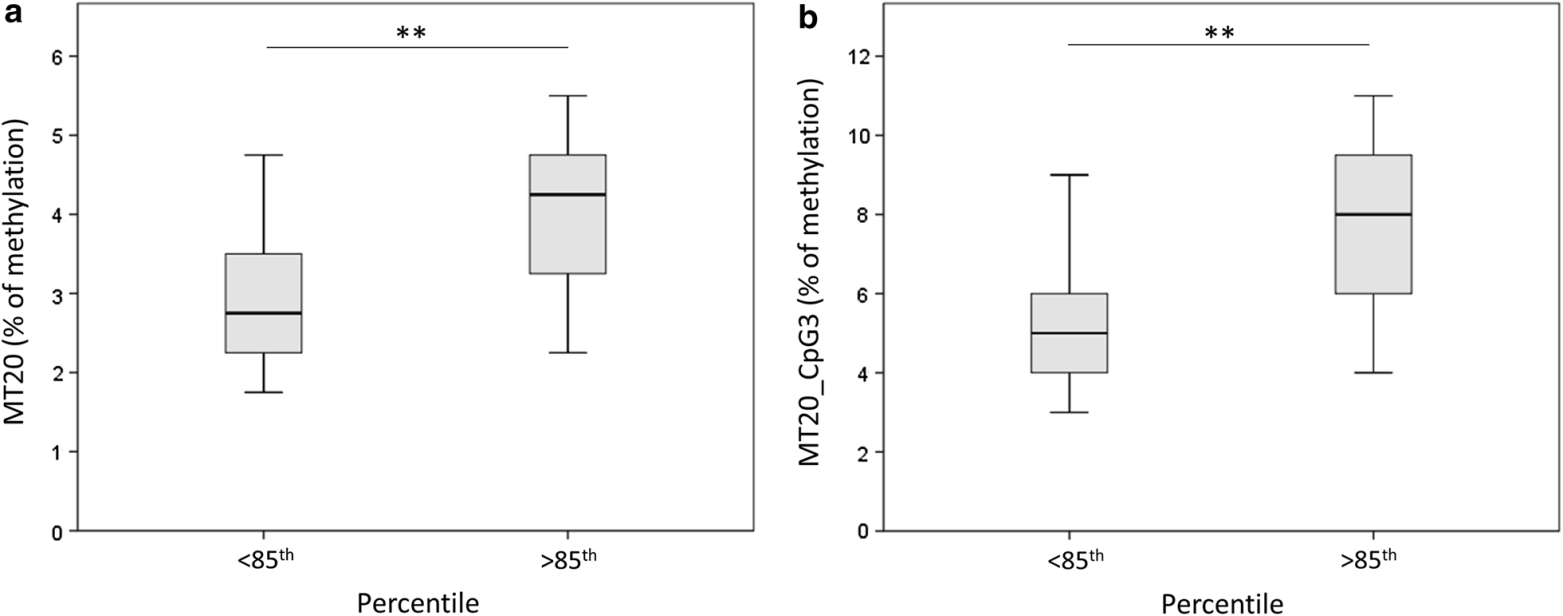
D-loop methylation in lean vs overweight subjects. Mean % values of methylation of the D-loop (**a**) or the single CpG3 (**b**) are represented by dividing the population in lean vs overweight subjects

By categorizing the population in lean or overweight basing on the percentile (> or < than 85th), it emerged that the mean % of methylation is significantly higher in overweight than in lean subjects (GLM, p = 0.003) (Fig. 3a). One of the CpGs (the 3rd in the MT20 sequence) resulted to be associated with overweight also singularly (GLM, p = 0.004), suggesting that it is the main contributor of this detected association with overweight (Fig. 3b).

**Fig. 4:**
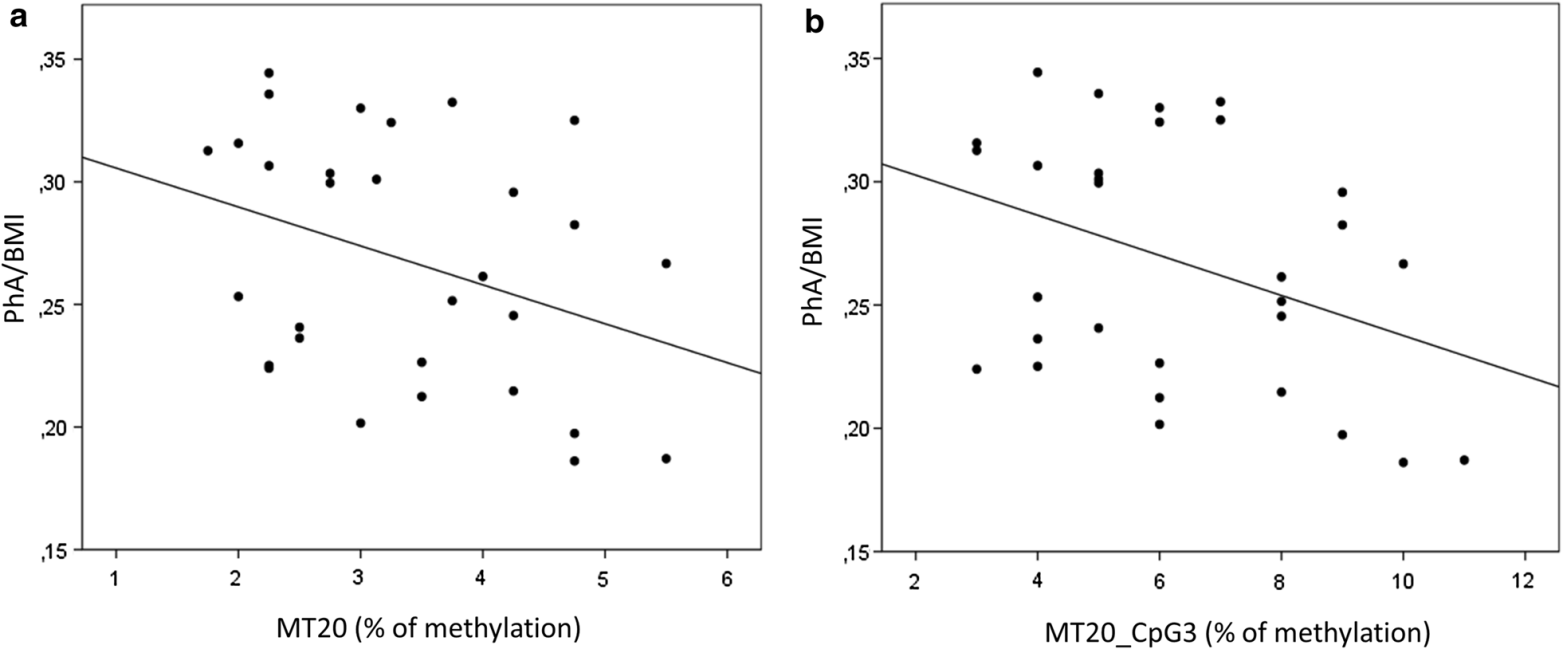
D-loop methylation and PhA/BMI ratio. Mean % values of methylation of the D-loop (**a**) or the single CpG3 (**b**) are plotted against the PhA normalized for BMI (PhA/BMI)

Concerning the continuous parameters describing the body composition, D-loop methylation at CpG3 resulted to be significantly associated to PhA/BMI (Pearson Correlation = − 0.374; p = 0.042) (Fig. 4a). A p for trend was calculated also testing correlation between the mean MT20% of methylation and PhA/BMI (Pearson Correlation = -0.351; p = 0.058) (Fig. 4b). No significant associations could be measured between D-loop methylation and the other parameters describing body composition.

### MtDNA copy number and D-loop methylation predict overweight in our female population

**Fig. 5:**
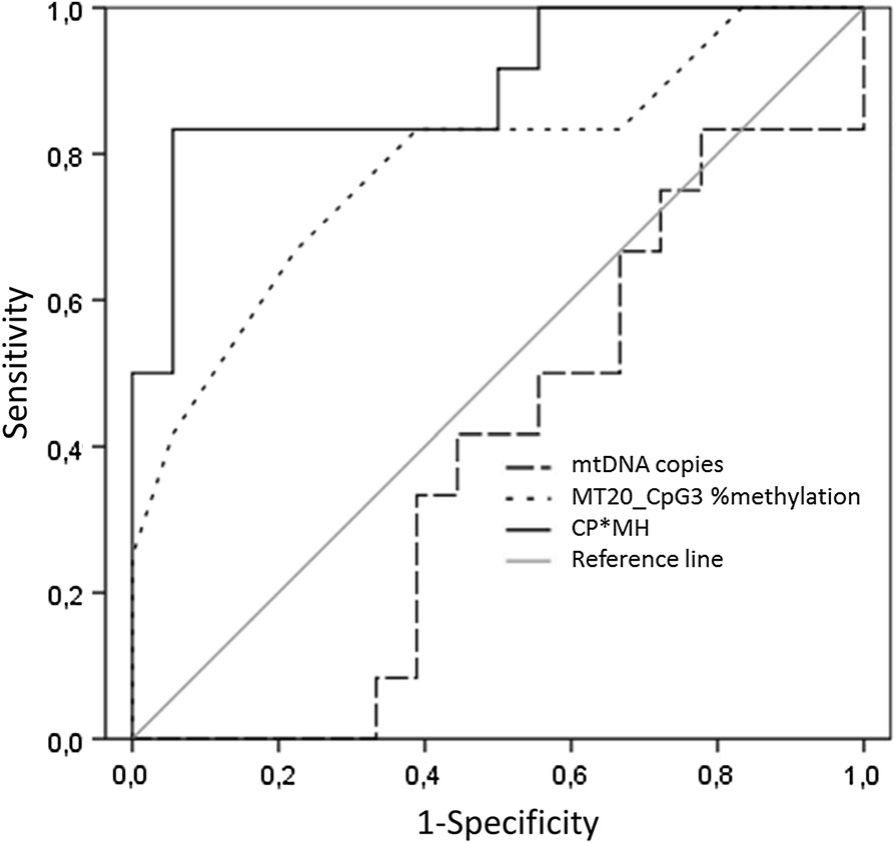
ROC curve predictions for overweight (percentile > 85th). AUC for the MtDNA copies, MT20_CpG3 methylation % and CP*MH variables are plotted

Testing the ability of mtDNA copy number and MT20_CpG3 methylation to predict overweight, ROC curve analysis showed that MT20_CpG3 methylation alone significantly predicts the overweight in our population (AUC = 0.785; p = 0.009), while the mtDNA copy number is not able to identify the overweight individuals from the others (AUC = 0.389; p > 0.05). The combined predicted probability (CP*MH) calculated from both the two variables (mtDNA copy number and CpG3 methylation) improved the prediction of the model for the overweight (AUC = 0.894; p = 0.00001), suggesting that the combination of these two variables could be more informative than the single ones (Fig. 5).

**Fig. 6:**
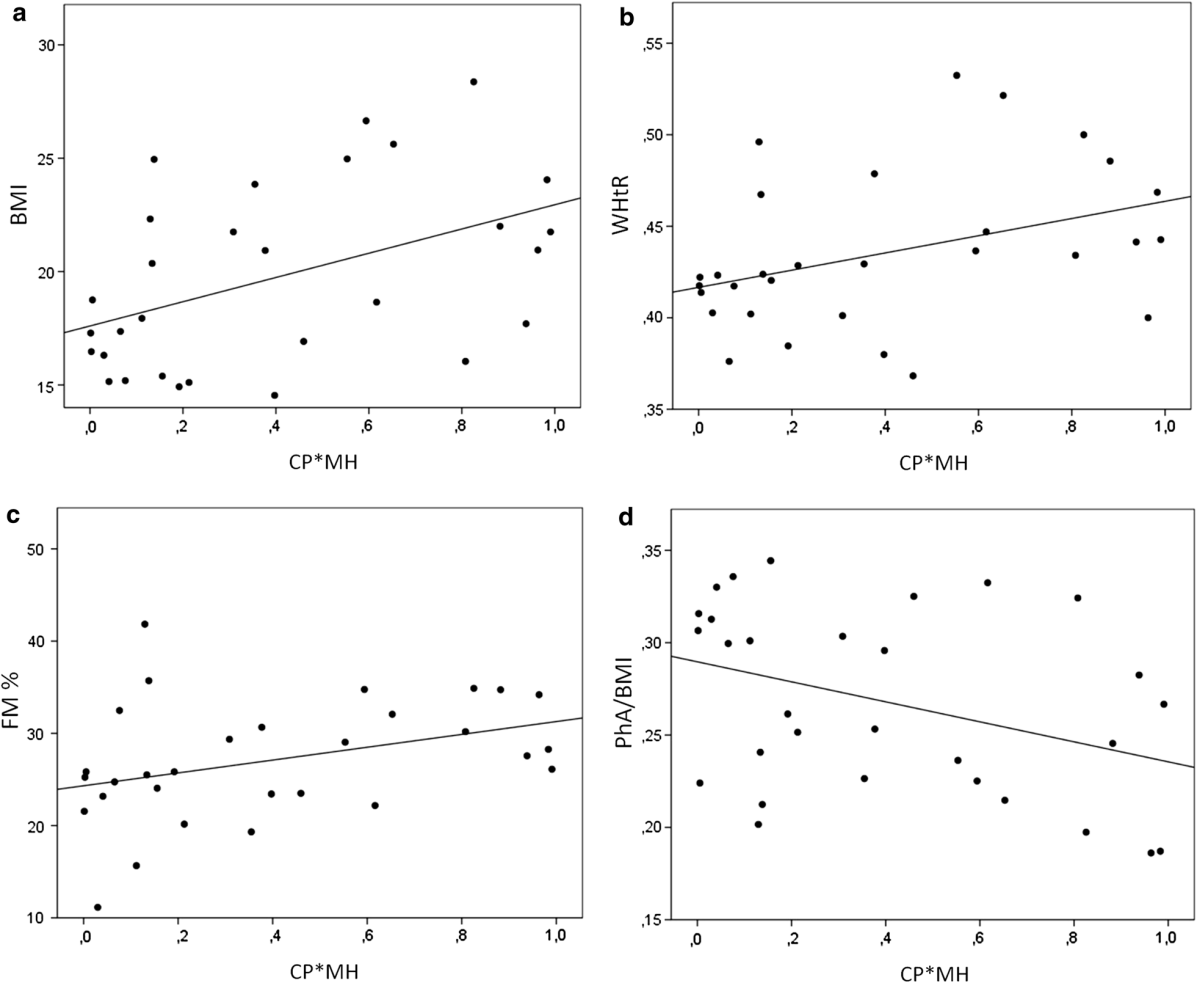
Association between CP + MH and changes in body composition. Scatter plot describing the correlation between CP*MH and BMI (**a**), WHtR (**b**), FM % (**c**) or PhA/BMI (**d**)

This hypothesis is supported by the evidence showing that this combined variable (CP*MH) linearly correlates to BMI (Spearman correlation = 0.438; p = 0.015) (Fig. 6a), WHtR (Spearman correlation = 0.435; p = 0.016) (Fig. 6b), FM % (Spearman correlation = 0.413; p = 0.023) (Fig. 6c), and negatively correlates with PhA/BMI (Spearman correlation = − 0.367; p = 0.046) (Fig. 6d). This means that this combined variable can better predicts body composition than mtDNA methylation alone, in a young female population.

## Discussion and conclusions

The mtDNA plays a major role in numerous diseases, ranging from neurological [49] to cardiovascular [50], and it has been previously studied as a biomarker mostly from the genetic point of view. For example, a buccal swab approach has been used to examine mitochondrial dysfunction in children to screen mitochondrial disease before other clinical presentation onset [51]. The study of DNA copy number and, only recently, the study of methylation patterns displayed high potential as candidate biomarkers of exposure and disease, particularly in multifactorial pathologies such as obesity. Alteration of mtDNA copy number in obesity has been described in several studies that investigated this parameter in adipose tissue [52] or surrogate markers such as peripheral blood [53]. For instance, the reduction of mtDNA copy number in peripheral blood buffy coat cells has been associated with BMI [54] and insulin resistance [25], particularly in subjects with metabolic syndrome [27]. Moreover, it has been independently associated with visceral fat accumulation in healthy young adults [30]. Variation of mtDNA copy number and its dynamics have been detected also before and after bariatric surgery, with a gender-specific trend [32], and a decrease in mtDNA copy number in blood has been associated also to increased mtDNA copy number in subcutaneous adipose tissue and TNF-α production of people with high BMI [52]. This evidence suggests that different trends of mtDNA copy number variation can be observed in different tissues and that they are linked to inflammatory process, increasing the potential implication of this biomarker. Indeed, it has been demonstrated that peripheral blood mitochondrial DNA copy number could be a novel potential biomarker for diabetic nephropathy in type 2 diabetes patients, as it can predict complications of this degenerative pathology [26]. Nevertheless, the mechanistic link between mtDNA copy number and health is still unclear. As reduced mtDNA copy levels has been generally associated with poor health, it has been speculated that a reduction of this parameter could lead to detrimental effects through an impaired ATP production and mitochondrial gene-expression [55] or an alteration of the oxidative stress-response [56].

Not only blood has been used as a surrogate sample to study mtDNA copy number, but also buccal swabs and saliva [33, 57]. For example, it has been recently proved that child’s buccal cell mitochondrial DNA content alters the association between heart rate variability and recent air pollution exposure at school. Interestingly, particulate matter exposure was associated with lower heart rate variability only in subjects with low number of mtDNA copies (while the association was not observed in children with high mtDNA content) [58]. Indeed, screening of mtDNA copies in children buccal swabs and identifying factors (i.e. overweight) that could affect this parameter, could be also useful to categorize subjects more prone to unfavourable responses to environmental effects. Buccal swabs and saliva have been previously used to investigate also the methylome of ncDNA in obesity or overweight. The usage of DNA methylation measured in whole blood cells as a marker for less accessible tissues, that are directly involved in disease, has been already proved [59], and differences in methylation profiles of peripheral blood DNA in obesity have been already demonstrated [60]. Also, epigenetic data on buccal swabs suggests an association between the saliva methylome and BMI in adolescence [61]. Interestingly, it has been demonstrated that, despite epigenetic marks are tissue-specific, DNA methylation in saliva appeared more similar to patterns from some brain regions than methylation in blood [62] and some similarities in methylation profile in blood and buccal cells in non-imprinted loci has been identified [63]. Remarkably, a meta-analysis demonstrated that adiposity might influence DNA methylation, and perturbation of these epigenetic pattern could predict future development of type 2 diabetes; this suggests that alterations in DNA methylation are consequences of adiposity, rather than causes [13]. In this context, to investigate alterations of the epigenetic profile in overweight and obesity could help to elucidate physiopathological molecular mechanisms involved, characterizing different forms of obesity and predicting their complication. Nevertheless, evidences on mitochondrial DNA methylation alteration in overweight and obesity, particularly from buccal swab samples, are missing.

Our study shows that mtDNA methylation patterns can be studied in DNA samples from buccal swabs, and that they could be associated to the body composition in overweight children. This evidence has been demonstrated in the female population but not in the male one, supporting the gender-specific features already observed concerning mtDNA [30–32]. Girls having lower mtDNA copies and higher methylation levels at the D-loop in their mtDNA extracted from buccal swabs showed a worst body composition, not only in terms of BMI, but also analyzing BIVA parameters such as FM % and PhA. We showed that taking into account both mtDNA copy number and the D-loop methylation it is possible to predict the overweight of female subjects in the analyzed population. These data are coherent with previous evidence demonstrating that the reduction of mtDNA copy number in blood of obese humans is associated with insulin resistance and may arise from increased D-loop methylation, suggesting an insulin signaling-epigenetic-genetic axis in mitochondrial regulation [25].

Our preliminary results are remarkably because they support the usage of mtDNA methylation also in surrogate samples such as mtDNA extracted from buccal swabs, and represent a first evidence that mtDNA from buccal swabs is affected by alteration of body composition. Moreover, in this study, body parameters are described not only using indexes such as BMI (which is an informative index at population level but doesn’t describe body composition [64]) but also WHtR (which is used to discriminate total and central fat and cardiovascular risk factors associated with obesity in children [65]) and BIVA parameters, such as FM % and PhA, that really describe body composition, also in children and adolescents [66–68]. Another strength of this study is that all participants are young individuals, aged from 7 to 17 years old. As epigenetics and mtDNA copy number [53] are affected by aging, a population of subjects homogeneous for age is a good model to study the effect of adiposity/body composition on mtDNA methylation and to reduce other cofounding factors like medication or comorbidities, which are very common in adult obese patients. A limitation of this study is that we did not have information about sexual maturation of the recruited subjects, so we could not correct body composition data for this aspect. A second limitation is that the purity of isolated mtDNA was not absolute and some potential residuals of gDNA could be retained in the samples. However, it have to be considered that (1) NUMTs are rarely generated from the D-loop area [46], which is the area that has been analysed in this study; (2) as this study is not focused on genetic variants but on DNA methylation, eventual NUMTs contaminants would be reflected in marginal error in the percentage of methylation measured; (3) even in this case, the error would be equally distributed through all the samples. For all these reasons, we think that, even if occurring, potential residuals of NUMTs do not invalidate the results of this study.

Concluding, this preliminary evidence should be replicated in independent studies and further investigations able to assess the direction of this association and the functional mechanisms are warranted. Nevertheless, there is an undeniable need to improve understanding of molecular determinants of healthy and unhealthy obese. Thus, our study support the hypothesis that further studies clarifying the role of mtDNA epigenetics could help to identify a new biomarker (also from buccal swabs) which is a biosensor of exposure potentially useful to clarify molecular aspects of obesity complications’ onset and to stratify the risk of metabolic syndrome for obese individuals [69].

## Data Availability

All data generated or analysed during this study are included in this published article and its additional files.

## Abbreviations

BCM: body cell mass
BCMI: BCM index
BIA: Bioimpedance Analysis
BMI: body mass index
CP: copy number
FFM: free fat mass
FM: fat mass
MH: methylation
mtDNA: mitochondrial DNA
ncDNA: nuclear DNA
PhA: phase angle
WC: waist circumference
WHtR: waist to height ratio
